# Interventions targeted at primary care practitioners to improve the identification and referral of patients with co-morbid obesity: a realist review protocol

**DOI:** 10.1186/s13643-015-0046-y

**Published:** 2015-05-01

**Authors:** David N Blane, Sara Macdonald, David Morrison, Catherine A O’Donnell

**Affiliations:** General Practice and Primary Care, Institute of Health and Wellbeing, University of Glasgow, 1 Horselethill Road, Glasgow, G12 9LX UK; Public Health, Institute of Health and Wellbeing, University of Glasgow, 1 Lilybank Gardens, Glasgow, G12 8RZ UK

**Keywords:** Realist review, Realist synthesis, Obesity, Primary care, General practitioners, Practice nurses, Referral, Weight management, Effectiveness

## Abstract

**Background:**

Obesity is one of the most significant public health challenges in the developed world. Recent policy has suggested that more can be done in primary care to support adults with obesity. In particular, general practitioners (GPs) and practice nurses (PNs) could improve the identification and referral of adults with obesity to appropriate weight management services. Previous interventions targeted at primary care practitioners in this area have had mixed results, suggesting a more complex interplay between patients, practitioners, and systems. The objectives of this review are (i) to identify the underlying ‘programme theory’ of interventions targeted at primary care practitioners to improve the identification and referral of adults with obesity and (ii) to explore how and why GPs and PNs identify and refer individuals with obesity, particularly in the context of weight-related co-morbidity. This protocol will explain the rationale for using a realist review approach and outline the key steps in this process.

**Methods:**

Realist review is a theory-led approach to knowledge synthesis that provides an explanatory analysis aimed at discerning *what works*, *for whom*, in *what circumstances*, *how*, and *why*. In this review, scoping interviews with key stakeholders involved in the planning and delivery of adult weight management services in Scotland helped to inform the identification of formal theories - from psychology, sociology, and implementation science - that will be tested as the review progresses. A comprehensive search strategy is described, including scope for iterative searching. Data analysis is outlined in three stages (describing context-mechanism-outcome configurations, exploring patterns in these configurations, and developing and testing middle-range theories, informed by the formal theories previously identified), culminating in the production of explanatory programme theory that considers individual, interpersonal, and institutional/systems-level components.

**Discussion:**

This is the first realist review that we are aware of looking at interventions targeted at primary care practitioners to improve the weight management of adults with obesity. Engagement with stakeholders at an early stage is a unique feature of realist review. This shapes the scope of the review, identification of candidate theories and dissemination strategies. The findings of this review will inform policy and future interventions.

**Systematic review registration:**

PROSPERO CRD42014009391

**Electronic supplementary material:**

The online version of this article (doi:10.1186/s13643-015-0046-y) contains supplementary material, which is available to authorized users.

## Background

### Rationale for the review

Obesity is widely regarded as one of the most significant public health challenges in the developed world [1]. Obesity is a risk factor for, amongst others, coronary heart disease, diabetes, stroke, osteoarthritis, and a number of different cancers [2] and is, ultimately, associated with premature death [3]. The benefits of weight loss for adults with obesity include reduced progression to type 2 diabetes [4,5] and lower blood pressure and cholesterol [6].

Strategies to prevent and treat obesity include interventions aimed at the individual, family, healthcare provider, and environmental levels [1]. Current UK and Scottish guidelines on obesity emphasise the central role of primary care (particularly general practitioners and practice nurses) in its prevention and management [7,8]. The strengths of primary care - population coverage, first contact, continuity, and relationships of trust built over serial encounters [9] - support this role in theory, but there is a considerable gap between policy rhetoric (‘every healthcare contact is a health improvement opportunity’ [10]) and the reality in practice. Obesity remains under-treated in primary care: few are referred to external sources of support, where they exist, and there are wide variations in referral rates and attendance following referral [11,12].

Qualitative research offers several proposed explanations for this sub-optimal engagement with weight management by general practitioners (GPs) and practice nurses (PNs), including lack of time in the consultation [13], lack of knowledge, and lack of confidence in discussing weight [14], perceptions of poor outcomes of interventions [14], and fear of causing offence [15]. We will briefly explore each of these in turn.

Time constraints are clearly an issue. For instance, a UK review recommending motivational interviewing as an evidence-based strategy to support weight loss for patients in primary care noted that 15 min was the minimum time found to be effective and training in motivational interviewing itself takes at least 2 days [16]. This is a time commitment that most GPs and PNs would be unable to make. More recently, attention has turned towards the use of brief interventions for weight management in primary care, building on the successful use of brief interventions for smoking cessation or alcohol [17]. It remains to be seen how transferrable such an approach might be in the context of weight management, as there are significant qualitative differences between the discussion of a patient’s weight and that of their smoking or alcohol intake, as the following barriers demonstrate.

A recent report from the Royal College of Physicians stated that training for GPs in weight management has been minimal and poorly coordinated, ‘reflecting a lack of focus on obesity throughout medical training as a whole’ [18]. Lack of knowledge and lack of confidence are far more pertinent in primary care weight management than they are for smoking or alcohol. Successful interventions to improve weight management in primary care are likely, therefore, to require at least an element of training for primary care practitioners, in particular GPs and practice nurses.

A further barrier is that many GP/PNs do not believe there are any effective interventions for patients with obesity and are, therefore, reluctant to refer patients to a service they believe will be ineffective [14]. Weight management interventions to date have had mixed results, but a growing number of studies have shown that primary care-based weight management programmes can be effective at achieving weight loss of ≥5 kg [19-21], a widely accepted weight loss target [8]. The issue is perhaps more accurately framed as being one of re-setting expectations - away from a target of ‘normal weight’ and towards a more realistic target of 5 to 10 kg weight loss (or 5 to 10% of body weight), or even healthy behaviour change (that is healthier diet and increased physical activity) regardless of weight loss.

Additional factors that may influence GP/PN engagement with weight management include attitudes to obesity (weight bias has been observed in many health professional groups [22,23]) and the weight of the health professional themselves. An overweight/obese practitioner may be less inclined to give advice on weight management [24]; similarly, an overweight/obese patient may be less inclined to take heed of any advice coming from a practitioner of similar weight.

Finally, fear of causing offence is another barrier to GP/PN engagement with weight management. Many GPs feel uncomfortable with the idea of raising a patient’s weight as a health issue if this is not the reason why the patient has attended. There is a significant social stigma associated with obesity, arguably more so than with smoking or alcohol, and GPs may be wary of upsetting a good doctor-patient relationship.

Overall, it is clear that any intervention targeted at primary care practitioners to improve weight management will have a number of potential barriers to consider. A Cochrane systematic review assessed the effectiveness of interventions to change the behaviour of health professionals and/or the organisation of care to promote weight reduction in overweight and obese people, using a search to May 2009 [25]. The review identified six RCTs, but only one of these was set in UK primary care [26]. It found evidence of a change in clinicians’ behaviours after receiving an educational intervention, but no statistically significant difference in patient weight between intervention and control groups, suggesting a more complex interplay between patients, practitioners, and systems.

A more recent systematic review (from 2011) found there were no trials examining the effect of primary care screening to identify overweight or obesity in adults and brief intervention was effective [27]. This review did, however, have restrictive inclusion criteria (only looking at RCTs), and it is likely that more recent research does exist in this area.

### Objectives and focus of the review

In keeping with policy recommendations, which emphasise the role of primary care in the *identification* of individuals with obesity and appropriate *signposting*/*referral* to services, the focus of this review is on how GPs and practice nurses identify individuals with obesity and why they refer some patients but not others.

This review will have a particular focus on the management of individuals with ‘high risk’ co-morbid obesity - that is obesity with weight-related co-morbidities, such as diabetes and hypertension. This focus is for two reasons: first, given the high prevalence of obesity, most health systems have adopted a tiered approach to weight management services, based on clinical need, as there is no capacity to see all individuals with obesity. Secondly, given the rising rates of co-morbid obesity, this is something that GPs and PNs will be seeing more of and must become better at managing.

The objectives of this review are (i) to identify the underlying ‘programme theory’ of interventions targeted at primary care practitioners to improve the *identification* and *referral* of adults with obesity and (ii) to explore *how* and *why* GPs and PNs *identify* and *refer* individuals with obesity, particularly in the context of weight-related co-morbidity.

### Review questions

1. What is the ‘programme theory’ of interventions targeted at primary care practitioners to improve the identification and referral of adults with obesity?
2. What are the mechanisms at play in different components of these interventions and what are the contextual factors that enable these mechanisms to produce successful outcomes?
3. How do GPs and practice nurses identify individuals with obesity?
4. Why do they refer some patients but not others?
5. What is the influence of co-morbidity on referral decisions?

## Methods

A realist approach was chosen for this review because it is particularly suited to the management of a mixed body of evidence, typical of complex interventions with multiple interacting components. Realist review or synthesis (RS) is based on the recognition that the same policy or intervention may be effective in one setting but completely ineffective in another. It provides an explanatory analysis aimed at discerning *what works*, *for whom*, in *what circumstances*, *how*, and *why* [28]. The ultimate ambition of a realist review is to produce (and test) a refined ‘programme theory’, which explains how and why a particular programme or intervention works (or not). It does this by applying a realist philosophy of causation and focussing, not on the intervention itself but on the mechanisms (M) that lead to successful - or unsuccessful - outcomes (O) in different contexts (C). It is expected that a realist synthesis will produce a *description* of positive and negative context-mechanism-outcome (CMO) configurations, explore patterns among these CMO configurations, and develop and test one or more middle-range theories that *explain* how and why these configurations relate to each other, drawing on formal theory (that is substantive theory from disciplines such as sociology, economics, psychology, and so on) in the domain in which the review is situated [29].

Unlike more conventional systematic reviews, there has, until relatively recently, been little guidance on how to conduct a realist synthesis. Rycroft-Malone and colleagues addressed this gap by providing a step-by-step account of a realist synthesis exploring the effectiveness of interventions to promote evidence use in health care [30]. More recently, Wong and colleagues produced the RAMESES quality standards for researchers undertaking realist reviews [29] and also publication standards for realist syntheses, which set out a 19-point list of items to be included when reporting a realist synthesis [31]. These resources will be used to structure the realist synthesis process in this project. There are several steps outlined in the quality standards that are considered to be good practice when conducting realist review, including scoping interviews, identification of candidate programme and formal theories, and iterative literature searching. These will be addressed in turn now.

### Scoping interviews

One aspect of a realist synthesis that is different from a systematic review is the opportunity to include scoping interviews at an early stage. Scoping interviews with relevant stakeholders allow the researcher to understand different perspectives on how the policy or intervention in question is believed to operate in practice and can help to shape nascent programme theories. This stage of work can also help to shape what literatures are searched and how it is interrogated. Stakeholders can be interviewed again later in the review process to assess whether the review findings resonate with their experience in practice.

In the present study, scoping interviews have been conducted with individuals involved in the planning and delivery of adult weight management services across a number of different health boards in Scotland. It is beyond the scope of this protocol paper to present detailed analysis of these interviews, but they have been valuable not just in informing the identification of candidate theories (see next section) but also in understanding the variation in contextual factors across different regions, in terms of the way that weight management services are resourced, organised, accessed, and evaluated. The topic guide used for these interviews, informed by Pawson [32], can be found in an additional file (see Additional file 1).

### Identifying candidate programme and formal theories

In the realist approach, the primary ambition of the research synthesis is explanation building [33]. As noted by Pawson, ‘[t]he purpose is to articulate underlying programme theories and then to interrogate the existing evidence to find out whether and where these theories are pertinent and productive’ [33]. As such, many realist syntheses begin with a literature search for candidate programme theories, before exploring the primary research around the area of interest to test the pertinence of these theories [34,35]. This approach does not work so well, however, when the extent of primary research in the area of interest is limited or unknown or when there is considerable heterogeneity in the interventions involved [36].

Both of these issues are relevant in this review. First, as noted above, we know that two systematic reviews found very few interventions targeted at primary care practitioners to improve the screening and referral of adults with obesity [25,27], though these are now a number of years old, and this is clearly an area where new research is being produced on a regular basis [17,37]. Second, we know from similar research into interventions targeted at primary care practitioners to improve identification and referral in sensitive areas - in this case intimate partner violence screening [38] - that there are a number of different potential intervention components (for example effective protocols, ongoing training, feedback, improving access to support), which may in turn have different mechanisms underpinning them (for example practitioner self-efficacy, trust and confidence in the service, accepting responsibility).

It is possible that similar intervention strategies, perhaps with similar mechanisms, have been implemented in interventions related to weight management in primary care, but we have not attempted, at the start of the review process, to sketch out the various programme theories that may be at play across a potentially wide range of different interventions strategies. For instance, an intervention that aims to increase the identification of patients with obesity by providing desktop BMI calculators to primary care practitioners will have a very different programme theory to an educational intervention. As such, we made the decision to search for the interventions first and then to develop the programme theories underpinning these interventions. We have, however, identified a number of formal or substantive theories - from psychology, sociology, and implementation science - that we believe will be pertinent to this area of enquiry, through a two-stage process: (i) background reading and expert opinion and (ii) stakeholder interviews.

Background reading has been ongoing for some time, prior to the drafting of the proposal for the funding of this project. Expert opinion has been sought in the form of project supervisors, an advisory panel of academics, and presentation of research plans at interdisciplinary meetings and national conferences.

Stakeholder interviews, as noted above, were conducted with healthcare professionals, across Scotland, responsible for delivering weight management services that receive referrals from primary care. We sought the views of these professionals on how they engage with primary care practitioners, what they thought the barriers to identification and referral are and what they consider to be the most effective methods for increasing appropriate referrals. While few interviewees mentioned specific theories, several did draw attention to factors that influence the referral process at different levels (for example interpersonal versus institutional) and some were mindful of behaviour change theories.

This process has revealed three overlapping levels, within which potentially relevant theoretical fields are situated:

1. *Individual-level* theories of practitioner behaviour change (for example Theoretical Domains Framework [39,40], Behaviour Change Wheel [41])
2. *Interpersonal-level* theories of doctor-patient interaction (for example candidacy theory [42], theories of stigma [43], and shame [44])
3. *Institutional or system-level* theories of implementation (for example diffusion of innovations [45], normalisation process theory [46], PARiHS framework [47])

This is a somewhat artificial categorisation of theories into these different levels, as almost all of them operate to a greater or lesser extent across all three levels.

These substantive theories will be used to help make sense of the CMO patterns identified during the synthesis phase. It is important to note that these theories are likely to be refined in an iterative process as the review progresses and the programme theories of the interventions are developed.

### Search strategy

A comprehensive search strategy was developed in conjunction with the subject librarian of the University of Glasgow. This was adapted from the search strategy used by a previous Cochrane systematic review in the area [25], but with some important differences. First, search terms for different study designs were not included, as the realist approach does not exclude studies on the basis of design (for example qualitative studies can provide useful information on potential mechanisms). Secondly, the databases used were extended to include Ovid MEDLINE, EMBASE, CINAHL, PsycINFO, Web of Science, and ScienceDirect. Thirdly, the start date for the search was from 2004 onwards. This was the year the new General Medical Services (GMS) GP contract was implemented in the UK, when the Quality and Outcomes Framework (QOF) was introduced. QOF set important standards for record keeping and chronic disease/risk factor management, albeit in relation to performance monitoring and practice incentivisation. It was also the year of the UK government’s landmark White Paper, *Choosing Health: Making Healthier Choices Easier* [48], which signalled a shift of attention towards public health and overweight/obesity in particular. It also highlighted the role of healthcare practitioners in supporting people to make healthy lifestyle changes. (The search strategy can be found in Additional file 2.)

Citation searches and snowballing strategies will be used as the study progresses. Furthermore, results of stakeholder discussions and interviews, as well as training and conference material, may also be used as data.

### Iterative searching

As the synthesis progresses and nascent programme theories are developed and tested, further iterative searches will be necessary. This will involve purposive searching of the literature for evidence to support or refute candidate programme theories that are most relevant from the initial data analysis, potentially incorporating additional formal theories that may be identified during the review process.

### Study selection

Titles and/or abstracts of studies retrieved using the search strategy and those from additional sources will be screened independently by two review authors to identify studies that potentially meet the inclusion criteria. The questions and responses used for screening at title and abstract levels can be found in Table 1. This process will be conducted using Distiller SR software, with references uploaded from the EndNote reference manager software. Inclusion criteria at the title and abstract screening level will be broad, including any primary study (quantitative or qualitative) describing or reporting on an intervention, targeted at primary care practitioners, to improve the management of adults with obesity. Exclusion criteria will include non-English language, non-adult studies, review articles, and opinion pieces. Review articles will, however, be checked for relevant primary studies.

**Table 1:** **Screening questions and responses at title and abstract levels**

**Screening question**		**Response**
Title level	Could this be about adult weight management in primary care?	Yes
		No, not adult
		No, not weight management
		No, not primary care
		Unclear
Abstract level	Could this article provide useful information about the identification and referral of adults with obesity in primary care?	Yes
		No, not weight management for adults with obesity
		No, not involving primary care practitioners
		No, not related to identification or referral
		No, not original research (for example review, opinion piece, conference abstract)

The full text of these potentially eligible studies will be retrieved and independently assessed by two review team members. We recognise that double screening at title, abstract, and full paper levels is not a requirement for realist reviews, but we adopted this more rigorous approach as this review is part of DB’s PhD. Any disagreement between reviewers over the eligibility of particular studies will be resolved through discussion with a third reviewer. Reasons for exclusion will be documented.

### Data extraction and synthesis

A standardised, pre-piloted form will be used to extract data from the included studies for assessment of study quality and evidence synthesis. Extracted information will be considered in terms of ‘context, mechanism, and outcome’ and will include study setting; study population, participant demographics and baseline characteristics; details of the intervention and control conditions (if appropriate); study methodology; recruitment and study completion rates; outcomes and times of measurement; indicators of acceptability to users; any suggestion by the authors of mechanisms of action of the chosen intervention strategies; and information for assessment of the risk of bias. Two review authors will extract data independently, and any discrepancies will be identified and resolved through discussion (with a third author where necessary).

In this review, we have adopted an approach similar to that taken by Jagosh and colleagues [36], of first searching for studies relating to interventions in this field, then developing initial programme theory, and then, after immersion in the data, reviewing the goodness of fit with pre-existing formal theory. A three-stage process of analysis will be followed.

In the first stage of analysis for each included study, a CMO configuration will be identified, describing how contextual factors interact with mechanisms to produce outcomes. Outcomes will include final desired outcomes, such as identification of obesity, recording of BMI, and referral to a weight management service, but also more proximal outcomes, such as markers of practitioner behaviour change (for example change in measures of knowledge or attitude) or system-level outcomes (for example improved communication between weight management service and practitioners) that make the final desired outcomes more probable. There are likely to be long implementation chains in any complex intervention, with each link in the chain having its own CMO configuration. Studies will be grouped together into those with similar intervention strategies, such that an initial programme theory or theories can be articulated.

The second stage of analysis will involve exploring patterns within these CMO configurations. Mechanisms will be compared across different contexts to assess if they are consistent in producing similar outcomes. In this way, statements of what works, for whom and in what circumstances (so-called demi-regularities) can be formulated.

The final stage of analysis will involve configuring these demi-regularities into a coherent and plausible ‘refined’ programme theory, drawing on the formal theory previously identified. It is anticipated that, through the process of increasing familiarity with the data, a shortlist of the most apposite theories from the initial scoping search will be determined and the empirical data used to test and refine the ‘best fit’ theory. It is hoped that an explanatory programme theory (or theories) encompassing individual, interpersonal, and institutional/systems-level components will be produced.

It is likely that, in some cases, study authors will be contacted to obtain further information on some aspects of their study. For example, contextual and implementation factors are often poorly described in journal articles due in part to word limits.

### Quality appraisal

In realist synthesis, studies are assessed based on two criteria: relevance - whether they contribute to theory building and/or testing and rigour - whether the methods used to generate the relevant data are credible and trustworthy. We will, however, also assess the quality of the included studies to develop an understanding on the quality and rigour of the evidence underpinning our theory development. For qualitative studies, we will use Popay *et al*. [49]; for other study types, we will use CASP checklists. Studies will not, however, be routinely excluded on the basis of quality appraisal.

### Reporting and dissemination of findings

In addition to producing a report for the funders of this review, a paper will be submitted to a leading journal in this field. The RAMESES reporting standards will be followed. Furthermore, findings will be disseminated through consultations with stakeholders. The lead researcher (DB) is already in communication with key stakeholders within the local health board and the regional weight management service. Should the findings of the review warrant a change in practice, a one-page summary report will be prepared and sent to lead clinicians and healthcare professionals in the National Health Service in Scotland.

### Ethical issues

Ethical approval for stakeholder interviews was obtained from the University of Glasgow College of Medical, Veterinary and Life Sciences Ethics Committee [*Project No:* 200130121]. The study protocol has been registered with PROSPERO, the international prospective register of systematic reviews: CRD42014009391.

## Discussion

### Limitations

This realist review has a number of limitations. Firstly, as has been widely reported in other realist reviews, many study authors do not describe contextual factors in detail or discuss the mechanisms that explain their study outcomes. This will be addressed by contacting authors for clarification and by asking for any related reports that may provide additional contextual information. A further limitation is the risk of selective bias by searching the literature for relevant interventions in the first instance and then developing programme theory and applying formal theoretical frameworks thereafter. This was a decision made in a transparent manner from the outset, due to uncertainty around the number and heterogeneity of studies in this area.

### Summary

Obesity is widely regarded as one of the most significant public health challenges of our time. Recent policy suggests that primary care practitioners (that is GPs and practice nurses) could do more in the identification and referral of adults with obesity. This review of previous interventions in this area uses a realist synthesis methodology to develop and test the programme theory of these interventions, through exploration of context-mechanism-outcome configurations of successful (and unsuccessful) interventions. This protocol has outlined key features of realist synthesis, including the use of stakeholder interviews, identifying candidate theories, and the iterative nature of searching. The process of data extraction, quality appraisal, and analysis is also described. Stakeholders will be consulted again as the review progresses to ensure the findings resonate with their experience.

## Additional files

Additional file 1:
**Stakeholder interview topic guide.** This file contains the theory-driven topic guide used for the stakeholder interviews. Additional file 2:
**Search strategy.** This file describes the initial search strategy undertaken on six databases.

## Abbreviations

BMI: body mass index
CASP: Critical Appraisal Skills Programme
CMO: context-mechanism-outcome
GMS: general medical services
GP: general practitioner
PARiHS: Promoting Action on Research implementation in Health Services
PN: practice nurse
QOF: Quality and Outcomes Framework
RAMESES: Realist and Meta-narrative Evidence Syntheses - Evolving Standards
RCT: randomised controlled trial
RS: realist synthesis
SR: systematic review
