# Supplementary material for: Interventions targeted at primary care practitioners to improve the identification and referral of patients with co-morbid obesity: a realist review protocol
Source: Syst Rev. 2015 May 1;4:61. doi: 10.1186/s13643-015-0046-y (PMC4426175; doi:10.1186/s13643-015-0046-y)
Supplement: Additional file 1: — Stakeholder interview topic guide. This file contains the theory-driven topic guide used for the stakeholder interviews. [file 13643_2015_46_MOESM1_ESM.pdf]

# THEORY-DRIVEN STAKEHOLDER INTERVIEWS TOPIC GUIDE

**Intro:** Make it clear who I am, why I am there, and why I am asking *them*.

Go through consent, audio-recording, housekeeping

*In this realist review, the aim is to unpick interventions that have targeted primary care practitioners to improve the management of obesity, and I am particularly interested in co-morbid obesity. The focus is on potential mechanisms that might explain outcomes in certain contexts, e.g. why do some GPs refer more than others, and why are some people referred more than others?*

**<u>Interviewee background</u>**

- I wonder if I can start by asking you to introduce yourselves - What is your current role? What involvement have you had with weight management services in primary care?

- Could you tell us a bit more about the weight management services in [your NHS region]?
    - Tier 2 – NHS or local authority?
    - Tier 3
    - Access to bariatric surgery?

- Do you monitor referrals by GPs?
- Do you meet with other weight management services?

**<u>Your experience of primary care weight management</u>**

- Have you had experience of engaging with primary care practitioners with regard to weight management?
- Have you found it easier to engage with GPs or PNs?
- What have you found to be the most effect *methods* of engagement, e.g. letter, email, meetings, open days, visiting practices, etc?

**<u>Role of Primary care in weight management</u>**

- What do you think the role of GPs should be in weight management?
- What do you think the role of PNs should be in weight management?
- To what extent do you think these roles are being fulfilled currently?
- Did you have any experience of Counterweight?

- How is weight management funded where you are?

*Thinking about the outcomes of **increased identification** and **referral** of patients with obesity to weight management services…*

- Do you think there are any differences in these outcomes by *practitioner characteristics*, e.g. age/experience, gender, BMI
- Are there differences in these outcomes by *practice characteristics*, e.g. size, location (urban/rural; deprived/affluent), teaching/training practice
- Are some patients more likely to be referred that others? E.g. *patient characteristics* such as age, gender, SES, rurality?
- Are patients with certain **co-morbidities** more or less likely to be referred?
    - Depression
    - Diabetes
    - Mechanical joint pain

*Previous research has suggested a number of possible explanations why GPs **do not identify** (and record) patients as having obesity. What do you think?*

*Similarly, there are a number of possible explanations why GPs **do not refer** more patients with obesity to weight management services. What do you think?*

**Future interventions**

- How would you approach improving the **identification** of individuals with obesity in primary care?

- How would you approach improving the **referral** of individuals with obesity in primary care to weight management services?

**Any other comments or suggestions for changes to future stakeholder interviews?**

**Many thanks for taking part**
