# Supplementary material for: Interventions targeted at primary care practitioners to improve the identification and referral of patients with co-morbid obesity: a realist review protocol
Source: Syst Rev. 2015 May 1;4:61. doi: 10.1186/s13643-015-0046-y (PMC4426175; doi:10.1186/s13643-015-0046-y)
Supplement: Additional file 2: — Search strategy. This file describes the initial search strategy undertaken on six databases. [file 13643_2015_46_MOESM2_ESM.pdf]

Additional file 1

Search strategy

OVID MEDLINE

1. exp Obesity/ or exp Obesity, Morbid/
2. (obes$ or overweight$).tw.
3. Weight Loss/
4. 1 or 2 or 3
5. exp Education, Continuing/
6. (education$ adj2 (program$ or intervention? or meeting? or session? or strateg$ or workshop? or visit?)).tw.
7. (behavio?r$ adj2 intervention?).tw.
8. *pamphlets/
9. (leaflet? or booklet? or poster or posters).tw.
10. ((written or printed or oral) adj information).tw.
11. (information$ adj2 campaign).tw.
12. (education$ adj1 (method? or material?)).tw.
13. outreach.tw.
14. ((opinion or education$ or influential) adj1 leader?).tw.
15. facilitator?.tw.
16. Practice Guideline as Topic/
17. *guideline adherence/
18. practice guideline?.tw.
19. (guideline? adj2 (introduc$ or issu$ or impact or effect? or disseminat$ or distribut$)).tw.
20. ((effect? or impact or evaluat$ or introduc$ or compar$) adj2 training program$).tw.
21. *reminder systems/
22. reminder?.tw.
23. (recall adj2 system$).tw.
24. (prompter? or prompting).tw.
25. *feedback/ or feedback.tw.
26. chart review$.tw.
27. ((effect? or impact or records or chart?) adj2 audit).tw.
28. compliance.tw.
29. marketing.tw.
30. or/5-29
31. exp Patient Care Team/
32. exp Primary Health Care/
33. exp Family Practice/ or exp General Practice/
34. exp *Health Personnel/
35. (team? adj2 (care or treatment or assessment or consultation)).tw.
36. (integrat$ adj2 (care or service?)).tw.
37. (care adj2 (coordinat$ or program$ or continuity)).tw.
38. (case adj1 management).tw.
39. *ambulatory care/
40. or/31-39
41. exp "Referral and Consultation"/
42. ((effect? or impact or evaluat$ or introduc$ or compar$) adj2 (treatment or care or screen$ or prevent$) adj2 program$).tw.
43. ((effect? or impact or introduc$) adj2 (legislation or regulations or policy)).tw.
44. *medical records/
45. *medical records systems, computerized/
46. (information adj2 (management or system?)).tw.
47. *utilization review/
48. *Quality Assurance, Health Care/
49. Quality of Health Care/
50. *program evaluation/
51. triage.tw.
52. *telephone/

53. (physician patient adj (interaction? or relationship?)).tw.
54. ((standard or usual or routine or regular or traditional or conventional or pattern) adj2 care).tw.
55. (program$ adj2 (reduc$ or increas$ or decreas$ or chang$ or improv$ or modify$ or monitor$ or care)).tw.
56. ((introduc$ or impact or effect? or implement$ or computer$) adj protocol?).tw.
57. (computer$ adj2 (diagnosis or decision?)).tw.
58. or/41-57
59. 30 or 58
60. 4 and 40 and 59
61. animal/
62. human/
63. 61 not (61 and 62)
64. 60 not 63
65. Child/
66. 64 not 65
67. limit 66 to (english language and yr="2004 -Current")

EMBASE (OVID)

1. exp Obesity/
2. (obes$ or overweight$).tw.
3. weight reduction/
4. or/1-3
5. exp medical education/
6. (education$ adj2 (program$ or intervention? or meeting? or session? or strateg$ or workshop? or visit?)).tw.
7. (behavio?r$ adj2 intervention?).tw.
8. publications/
9. medical information/
10. information dissemination/
11. information service/
12. (leaflet? or booklet? or poster or posters).tw.
13. ((written or printed or oral) adj information).tw.
14. (information$ adj2 campaign).tw.
15. (education$ adj1 (method? or material?)).tw.
16. outreach.tw.
17. ((opinion or education$ or influential) adj1 leader?).tw.
18. facilitator?.tw.
19. consensus conference?.tw.
20. exp Practice Guideline/
21. practice guideline?.tw.
22. (guideline? adj2 (introduc$ or issu$ or impact or effect? or disseminat$ or distribut$)).tw.
23. ((effect? or impact or evaluat$ or introduc$ or compar$) adj2 training program$).tw.
24. reminder system/
25. reminder?.tw.
26. decision support system/
27. (recall adj2 system$).tw.
28. (prompter? or prompting).tw.
29. *feedback/ or feedback.tw.
30. chart review$.tw.
31. ((effect? or impact or records or chart?) adj2 audit).tw.
32. compliance.tw.
33. marketing.tw.
34. or/5-33
35. patient care/
36. patient care planning/
37. general practice/

38. general practitioner/
39. nurse practitioner/
40. (team? adj2 (care or treatment or assessment or consultation)).tw.
41. (integrat$ adj2 (care or service?)).tw.
42. (care adj2 (coordinat$ or program$ or continuity)).tw.
43. (case adj1 management).tw.
44. case management/
45. exp primary healthcare/
46. *ambulatory care/
47. healthcare practice/
48. community health center/
49. healthcare facility/
50. *group practice/
51. medical practice/
52. or/35-51
53. *medical record/
54. (information adj2 (management or system?)).tw.
55. "peer review"/
56. "utilization review"/
57. clinical practice/
58. quality assurance.tw.
59. Outcome Assessment/
60. Total Quality Management/
61. Health Care Quality/
62. program evaluation/
63. triage.tw.
64. patient referral/
65. *telephone/
66. (physician patient adj (interaction? or relationship?)).tw.
67. *health maintenance organizations/
68. managed care.tw.
69. or/53-68
70. ((standard or usual or routine or regular or traditional or conventional or pattern) adj2 care).tw.
71. (program$ adj2 (reduc$ or increas$ or decreas$ or chang$ or improv$ or modify$ or monitor$ or care)).tw.
72. ((effect? or impact or evaluat$ or introduc$ or compar$) adj2 (treatment or care or screen$ or prevent$) adj2 program$).tw.
73. (computer$ adj2 (diagnosis or decision?)).tw.
74. ((introduc$ or impact or effect? or implement$ or computer$) adj protocol?).tw.
75. ((effect? or impact or introduc$) adj2 (legislation or regulations or policy)).tw.
76. or/70-75
77. 34 or 52 or 69
78. 4 and 77
79. nonhuman/
80. 78 not 79
81. 80
82. limit 81 to (english language and yr="2004 -Current")
83. child/
84. 82 not 83

Additional file 1

CINAHL (EBSCO)

1. (MH “Obesity+”) or (MM “Weight Loss”)
2. TX (obes* or overweight*)
3. 1 or 2
4. (MH “Education, Continuing+”) or (MM “Pamphlets”) or (MM “Practice Guidelines”) or (MM “Professional Compliance”) or(MM “Reminder Systems”)
5. TX (education* N2 (program*or intervention* or meeting* or session* or strateg* or workshop* or visit*)
6. TX (behavior* N2 intervention*) or TI (behaviour* N2 intervention*) or AB (behavior* N2 intervention*)
7. TX (leaflet* or booklet* or poster or posters)
8. TX (written information) or TX (printed information) or TX (oral information)
9. TX (information* N2 campaign)
10. TX (education* N1 method*) or TX (education* N1 material*)
11. TX (outreach) or TX (facilitator*)
12. TX (opinion N1 leader*) or TX (education* N1 leader*) or TX (influential N1 leader)
13. TX (practice guideline*)
14. TX (guideline* N2 (introduc*or issu* or impact or effect* or disseminat* or distribut*))
15. TX ((effect* or impact or evaluat* or introduc* or compar*) N2 training program*)
16. TX (reminder*) or TX (recall N2 system*) or TX (prompter*) or TX (prompting)
17. TX (chart review*)
18. TX ((effect* or impact or records or chart*) N2 audit)
19. TX (compliance) or TX (marketing)
20. 4 or 5 or 6 or 7 or 8 or 9 or 10 or 11 or 12 or 13 or 14 or 15 or 16 or 17 or 18 or 19
21. (MH “Health Personnel+”)
22. (MH “Multidisciplinary Care Team+”) or (MM “Ambulatory Care”)
23. (MM “Family Practice”) OR (MM “Physicians, Family”)
24. TX (“patient care planning” or “case management”
25. TX (integrat* N2 care) or TX (integrat* N2 service*)
26. TX (care N2 (coordinat* or program* or continuity))
27. TX (chang* N2 location*) or TX (home N2 treat*)
28. 21 or 22 or 23 or 24 or 25 or 26 or 27
29. (MH “Medical Records+”)
30. (MM “Peer Review”) or (MM “Utilization Review”)
31. (MM “Quality Assurance”) or (MM “Outcome Assessment”) or (MM “Quality Improvement”) or (MM “Quality of Health Care”) or (MM “Program Evaluation”) or (MH “Referral and Consultation+”) or (MM “Patient History Taking”) or (MM “Telephone”)
32. (MM “Process Assessment (Health Care)”)
33. TX (computer* N2 diagnosis) or TX (computer* N2 decision*)
34. TX ((standard or usual or routine or regular or traditional or conventional or pattern) N2 care)
35. TX (program*N2 (reduc* or increas* or decreas* or chang* or improv* or modif* or monitor* or care))
36. TX ((effect* or impact or evaluat* or introduc* or compar*) N2 “treatment program*”)
37. TX ((effect* or impact or evaluat* or introduc* or compar*) N2 “care program*”)
38. TX ((effect* or impact or evaluat* or introduc* or compar*) N2 “screening program*”)
39. TX ((effect* or impact or evaluat* or introduc* or compar*) N2 “prevention program*”)
40. TX ((introduc* or impact or effect* or implement* or computer*) N2 protocol*)
41. TX (effect* N2 (legislation or regulations or policy)) or TX (impact* N2 (legislation or regulations or policy)) or TX (introduc* N2 (legislation or regulations or policy))
42. TX (information N2 management) or TX (information N2 system*)
43. TX (“physician practice patterns”) or TX (“quality assurance”)
44. TX (“triage” or “managed care”)
45. TX (“physician patient interaction*”) or TX (“physician patient relationship*)
46. 29 or 30 or 31 or 32 or 33 or 34 or 35 or 36 or 37 or 38 or 39 or 40 or 41 or 42 or 43 or 44 or 45
47. 20 or 46
48. 3 and 28 and 47
49. Restrictions to year 2004 onwards and English language

Additional file 1

PsycINFO (EBSCO)

1. MJ Obesity or MJ Weight Loss
2. TX (obes* or overweight*)
3. 1 or 2
4. TX continuing education or TX physician education or TX Pamphlets or TX Practice Guidelines or TX Professional Compliance or TX Reminder Systems
5. TX (education* N2 (program*or intervention* or meeting* or session* or strateg* or workshop* or visit*)
6. TX (behavior* N2 intervention*) or TI (behaviour* N2 intervention*) or AB (behavior* N2 intervention*)
7. TX (leaflet* or booklet* or poster or posters)
8. TX (written information) or TX (printed information) or TX (oral information)
9. TX (information* N2 campaign)
10. TX (education* N1 method*) or TX (education* N1 material*)
11. TX (outreach) or TX (facilitator*)
12. TX (opinion N1 leader*) or TX (education* N1 leader*) or TX (influential N1 leader)
13. TX (practice guideline*)
14. TX (guideline* N2 (introduc*or issu* or impact or effect* or disseminat* or distribut*))
15. TX ((effect* or impact or evaluat* or introduc* or compar*) N2 training program*)
16. TX (reminder*) or TX (recall N2 system*) or TX (prompter*) or TX (prompting)
17. TX (chart review*)
18. TX ((effect* or impact or records or chart*) N2 audit)
19. TX (compliance) or TX (marketing)
20. 4 or 5 or 6 or 7 or 8 or 9 or 10 or 11 or 12 or 13 or 14 or 15 or 16 or 17 or 18 or 19
21. TX multidisciplinary practices OR TX ambulatory medical care
22. TX Health practitioners OR Health Personnel
23. TX family practice OR TX family physicians
24. TX (“patient care planning” or “case management”
25. TX (integrat* N2 care) or TX (integrat* N2 service*)
26. TX (care N2 (coordinat* or program* or continuity))
27. TX (chang* N2 location*) or TX (home N2 treat*)
28. 21 or 22 or 23 or 24 or 25 or 26 or 27
29. TX medical records
30. TX peer review OR TX utilization review
31. TX Quality Assurance OR TX Outcome Assessment OR TX Quality Improvement OR TX Quality of Health Care OR TX Program Evaluation OR TX Referral OR TX Telephone
32. TX Process Assessment
33. TX (computer* N2 diagnosis) or TX (computer* N2 decision*)
34. TX ((standard or usual or routine or regular or traditional or conventional or pattern) N2 care)
35. TX (program*N2 (reduc* or increas* or decreas* or chang* or improv* or modif* or monitor* or care))
36. TX ((effect* or impact or evaluat* or introduc* or compar*) N2 “treatment program*”)
37. TX ((effect* or impact or evaluat* or introduc* or compar*) N2 “care program*”)
38. TX ((effect* or impact or evaluat* or introduc* or compar*) N2 “screening program*”)
39. TX ((effect* or impact or evaluat* or introduc* or compar*) N2 “prevention program*”)
40. TX ((introduc* or impact or effect* or implement* or computer*) N2 protocol*)
41. TX (effect* N2 (legislation or regulations or policy)) or TX (impact* N2 (legislation or regulations or policy)) or TX (introduc* N2 (legislation or regulations or policy))
42. TX (information N2 management) or TX (information N2 system*)
43. TX (“physician practice patterns”) or TX (“quality assurance”)
44. TX (“triage” or “managed care”)
45. TX (“physician patient interaction*”) or TX (“physician patient relationship*)
46. 29 or 30 or 31 or 32 or 33 or 34 or 35 or 36 or 37 or 38 or 39 or 40 or 41 or 42 or 43 or 44 or 45
47. 20 or 46
48. 3 and 28 and 47
49. Restrictions to year 2004 onwards and English language

Additional file 1

Web of Science

1. **TOPIC:** (obesity) *OR* **TOPIC:** (weight management) *OR* **TOPIC:** (weight loss)
2. **TOPIC:** (family practice) *OR* **TOPIC:** (general practice) *OR* **TOPIC:** (primary care) *OR* **TOPIC:** (care NEAR/2 (coordinat* OR program* OR continuity)) *OR* **TOPIC:** (health personnel)
3. **TOPIC:** (medical education) *OR* **TOPIC:** (education NEAR/2 (program* OR intervention OR meeting OR session OR strateg* OR workshop OR visit))
4. **TOPIC:** (publications) *OR* **TOPIC:** ((written OR printed OR oral) NEAR/2 information) *OR* **TOPIC:** (information NEAR/2 campaign) *OR* **TOPIC:** (education NEAR/2 (method OR material))
5. **TOPIC:** (outreach) *OR* **TOPIC:** ((opinion OR education* OR influential) NEAR/1 leader) *OR* **TOPIC:** (facilitator) *OR* **TOPIC:** (practice guideline) *OR* **TOPIC:** (reminder) *OR* **TOPIC:** (decision support system) *OR* **TOPIC:** (recall NEAR/2 system)
6. **TOPIC:** (guideline NEAR/2 (introduc* OR issu* OR impact OR effect* OR disseminat* OR distribut*))
7. **TOPIC:** ((effect* OR impact OR evaluat* OR introduc* OR compar*) NEAR/2 training)
8. **TOPIC:** ((effect* OR impact OR records OR chart*) NEAR/2 audit) *OR* **TOPIC:** (feedback) *OR* **TOPIC:** (compliance) *OR* **TOPIC:** (marketing) *OR* **TOPIC:** (recall NEAR/2 system*)
9. **TOPIC:** (((effect* OR impact OR evaluat* OR introduc* OR compar*) NEAR/2 (treatment OR care OR screen* OR prevent*) NEAR/2 program*))
10. **TOPIC:** (outcome assessment) *OR* **TOPIC:** (program evaluation) *OR* **TOPIC:** (triage) *OR* **TOPIC:** (referral) *OR* **TOPIC:** ((physician AND patient NEAR/2 (interaction OR relationship))) *OR* **TOPIC:** (managed care)
11. **TOPIC:** ((program* NEAR/2 (reduc* OR increas* OR decreas* OR chang* OR improv* OR modify* OR monitor* OR care)))
12. **TOPIC:** (((effect* OR impact OR introduc* OR implement* OR computer*) NEAR/2 (treatment OR care OR screen* OR prevent*) NEAR/2 protocol*))
13. **TOPIC:** (((effect* OR impact OR introduc*) NEAR/2 (legislation OR regulations OR policy)))
14. **#3 OR #4 OR #5 OR #6 OR #7 OR #8 OR #9 OR #10 OR #11 OR #12 OR #13**
15. **#1 AND #2 AND #14**

Refined by: **RESEARCH AREAS:** NUTRITION DIETETICS OR HEALTH CARE SCIENCES SERVICES OR COMMUNICATION OR PSYCHOLOGY OR SOCIAL SCIENCES OTHER TOPICS OR EDUCATION EDUCATIONAL RESEARCH OR BEHAVIORAL SCIENCES OR SOCIOLOGY OR COMPUTER SCIENCE OR ANTHROPOLOGY OR MEDICAL ETHICS OR RESEARCH EXPERIMENTAL MEDICINE OR PUBLIC ADMINISTRATION

Restricted to year 2004 onwards and English language.

Science Direct

(obesity OR weight loss) AND (primary care OR general practice OR family practice) AND (refer* OR education* OR screen* OR feedback OR training OR guideline OR evaluat* OR effect* OR identif*)

Decision Sciences
Medicine and Dentistry
Nursing and Health Professions
Psychology
Social Sciences

Article
Review article
Short survey

2004 to date

Limit to 'topics' **"patient, weight loss, bariatric surgery, primary care, health care, life style, diabetes"**
